# Oral Cannabidiol for Acute Post-Extraction Pain: A Randomized Pilot Study

**DOI:** 10.3390/ph18121792

**Published:** 2025-11-25

**Authors:** Ammaar H. Abidi, Modar Kassan, Karen Derefinko

**Affiliations:** 1 College of Dental Medicine, Lincoln Memorial University, Knoxville, TN 37917, USA; modar.kassan@lmunet.edu; 2 College of Medicine, Department of Preventive Medicine, University of Tennessee, Memphis, TN 38163, USA

**Keywords:** cannabidiol (CBD), dental pain, tooth extraction, analgesia, non-opioid therapy, phytocannabinoids, acute postoperative pain, pilot clinical trial, pain management

## Abstract

**Introduction/Objective**: Dental extractions are among the most common oral surgical procedures worldwide, with postoperative pain representing a significant clinical concern. Cannabidiol (CBD), a non-intoxicating phytocannabinoid with analgesic and anti-inflammatory properties, has recently gained attention as a potential adjunct for managing acute dental pain. To explore its clinical utility to generate preliminary feasibility, we conducted the Simple Tooth Extraction with Analgesic Phytocannabinoid (SWAP) pilot trial to evaluate the preliminary efficacy and safety of oral CBD at two concentrations (17 mg/mL and 37 mg/mL) compared with placebo and standard ibuprofen/acetaminophen therapy following simple extractions. **Materials and Methods**: Eight adults were randomized equally to four arms (*n* = 2 per arm) CBD 17 mg/mL, CBD 37 mg/mL, placebo, or treatment-as-usual (TAU; ibuprofen/acetaminophen). CBD/placebo groups received 0.5 mL every 4–6 h as needed for 7 days, while TAU followed the non-opioid regimen. The primary endpoint was pain intensity (0–10 Numeric Rating Scale) captured via ecological momentary assessment (EMA) over 72 h. Secondary endpoints included worst pain, rescue medication use, adherence, tolerability, and qualitative feedback. **Results**: All participants completed follow-up with >75% EMA adherence. Because of the very small sample (*n* = 8), results are descriptive only. Baseline imbalance was observed; the CBD 17 mg/mL group reported substantially lower pre-extraction pain than other groups, limiting interpretability. Pain trajectories diverged by group: CBD 37 mg/mL showed the lowest ratings, paralleling TAU; CBD 17 mg/mL and placebo showed limited efficacy. **Conclusions**: This pilot suggests that higher-concentration CBD (37 mg/mL) may provide analgesia comparable to standard non-opioid therapy. Within this small feasibility cohort, higher-concentration CBD (37 mg/mL) appeared to produce pain patterns qualitatively similar to standard non-opioid therapy. Findings should be interpreted as exploratory only. A fully powered randomized trial incorporating biomarker endpoints and a taste-matched placebo is warranted.

## 1. Introduction

Dental extractions are among the most frequently performed oral surgical procedures worldwide, with postoperative pain being a common cause of unplanned follow-up visits. First-line pain management typically involves nonsteroidal anti-inflammatory drugs (NSAIDs) such as ibuprofen and naproxen, and acetaminophen (non-opioid analgesic), which are generally effective but may be limited by contraindications, adverse effects, or insufficient pain relief in some patients [1]. While generally effective, their prolonged or high-dose use of these medications may be associated with gastrointestinal irritation, peptic ulceration, hepatotoxicity, renal impairment, and cardiovascular risks [2,3]. Furthermore, a subset of patients either cannot tolerate these agents due to medical contraindications or do not achieve sufficient analgesia with standard dosing, creating a need for alternative or adjunctive non-opioid analgesic strategies with a favorable safety profile [3].

Cannabidiol (CBD) is a non-psychoactive phytocannabinoid derived from *Cannabis sativa* that has gained substantial research attention for its broad pharmacological properties, including analgesic, anti-inflammatory, anxiolytic, and neuroprotective effects [4,5,6,7,8]. Unlike Δ^9^-tetrahydrocannabinol (THC), CBD does not produce psychoactive effects [9] and has been investigated across multiple pain models, including postsurgical contexts, for its potential to reduce pain and inflammatory symptoms with a generally favorable tolerability profile [10,11,12].

The endocannabinoid system (ECS) is expressed in oral and periodontal tissues and contributes to immune regulation and tissue homeostasis [13]. In vitro, primary human gingival fibroblasts exposed to phytocannabinoids including CBD, cannabidivarin (CBDV), and cannabigerol (CBG) show reduced IL-1β–induced prostaglandin E2 and downregulation of pro-inflammatory cytokines (such as IL-6, IL-8) [4,14,15,16]. Although our trial did not include biomarker assays, these data support a plausible mechanism in which CBD attenuates inflammatory signaling, potentially limiting vascular permeability, edema, and nociceptor sensitization after extraction, an interpretation consistent with preclinical models of inflammatory and postsurgical pain.

Clinical data on CBD’s use for acute dental pain is limited. A randomized placebo-controlled clinical trial (*n* = 61) reported that a single oral CBD dose (10 or 20 mg/kg) produced significant pain relief within 180 min (up to 73% median visual analogue scale (VAS) reduction), with faster onset at 20 mg/kg and improved bite force; adverse effects were mostly mild (sedation, diarrhea, abdominal discomfort) and no psychoactive/mood changes were observed [17]. However, the mg/kg dosing may be impractical for routine dental care, highlighting the need to define clinically feasible regimens and long-term safety. Most other CBD analgesia studies address chronic conditions (e.g., neuropathic pain, MS-related spasticity, cancer pain) rather than acute post-extraction pain [18]. To date, there are no large, randomized trials directly comparing CBD with standard post-extraction regimens, leaving uncertainty about optimal dose, formulation, and risk-benefit in dental patients. Given CBD’s over-the-counter availability, rigorously designed trials are needed to generate evidence-based recommendations for acute dental pain management.

The present investigation was conceived before publication of the randomized clinical trial by Chrepa et al. [17] that evaluated single-dose oral CBD for acute dental pain. Our pilot differs by using concentration-based formulations (mg/mL), repeated dosing over seven days, and a real-world feasibility design aimed at refining procedures for a larger phase II trial.

Clinical data on CBD for acute dental pain are limited: Aside from a single-dose RCT in odontogenic pain, few trials address post-extraction settings, and no large studies compare CBD directly with standard non-opioid regimens. This leaves uncertainty around practical dosing, formulation, and safety in routine dental care. Given these gaps in the current literature, there remains a critical need for pragmatic, dental-specific investigations that evaluate CBD’s analgesic potential under real-world clinical conditions.

To address this, we designed the Simple Tooth Extraction with Analgesic Phytocannabinoid (SWAP) Study. SWAP evaluated the analgesic signal and safety of two oral CBD concentrations (17 mg/mL and 37 mg/mL) versus placebo and standard ibuprofen/acetaminophen in adults undergoing simple extractions. We prespecified that active treatments would show lower pain scores over the first 72 h than placebo, and explored whether CBD, particularly the higher concentration, could achieve analgesia comparable to standard therapy, with possible added benefit on inflammation suggested by prior laboratory data.

## 2. Results

### 2.1. Enrollment and Allocation

Before pandemic-related closure, 11 patients were consented; 8 met eligibility and were randomized equally across the four groups (*n* = 2 per arm: CBD37 mg/mL, CBD17 mg/mL, placebo, TAU). All randomized participants confirmed use of their assigned medication.

Table 1 shows descriptive information about race\, ethnicity, sex, education range, and average pain prior to surgery for the whole sample and for each medication group. Notably, the CBD17 mg/mL group demonstrated a low average pain prior to surgery (4 vs. 10 for all other groups).

### 2.2. EMA Compliance and Data Completeness

Table 2 shows EMA response rate. During the first 72 h, participants received up to 24 EMA prompts via text. Three groups achieved ≥75% completion (≥18 responses) meeting the predefined feasibility threshold, but the placebo group achieved only an average of 66.67% (16 responses). No early discontinuations or protocol deviations were noted.

### 2.3. Adherence

Day-7 bottle weights/pill counts indicated regular use in all arms. Descriptively, both CBD groups used oils consistently; the CBD17 group appeared to finish oil more frequently than placebo.

### 2.4. Pain Trajectories

**Figure 1:**
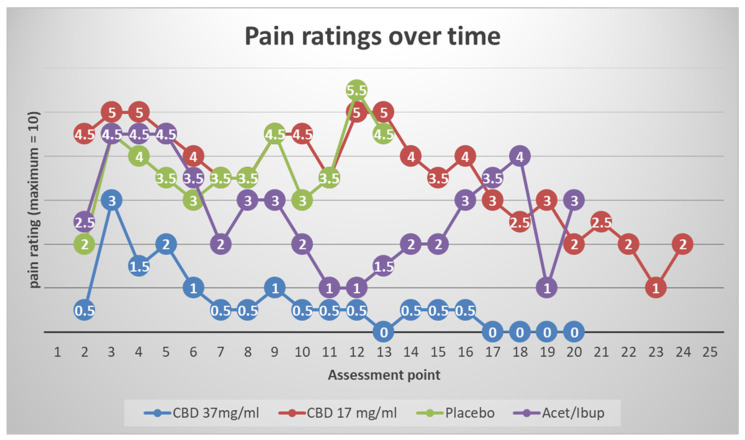
Time-series plots of Numeric Rating Scale (NRS) pain scores over 72 h post-extraction show qualitative separation by treatment. CBD 37 mg/mL produced the lowest and most stable pain ratings, paralleling treatment-as-usual (TAU; alternating acetaminophen/ibuprofen). CBD 17 mg/mL overlapped with placebo, which showed the highest and most prolonged pain scores. These trends suggest that higher CBD concentrations are required to achieve clinically meaningful analgesia after extraction. Plots depict individual trajectories; interpretations are descriptive rather than inferential because of the pilot sample size.

Figure 1 shows the time-series plots of NRS ratings suggested qualitative separation by condition:

CBD 37 mg/mL: The lowest and most stable pain ratings after the immediate post-operative window, with trajectories paralleling TAU (alternating acetaminophen/ibuprofen).

CBD 17 mg/mL: Pain ratings largely overlapped placebo across the 72-h window, with intermittent mid-range peaks (~4–5/10).

Placebo: The highest and most sustained pain scores, with peaks persisting beyond 48 h.

TAU: Low-to-moderate pain ratings, similar in shape to CBD37 mg/mL.

These descriptive patterns align with the priori expectation that a higher CBD concentration would be required to produce clinically visible analgesia in the acute post-extraction setting.

### 2.5. Blinding Integrity and Qualitative Feedback

Sensory cues and onset expectations influenced perceived assignment:

Taste/palatability: CBD 37 mg/mL was variably described as “horrible/fish oil” or “honey.” One participant in the CBD 17 mg/mL group described the taste as “very good and a bit like grapefruit.”. One placebo participant reported that they “did not have a taste.”

Onset expectations: Several participants in the CBD arms reported that pain did not go away immediately after dosing, leading them to suspect an inactive product.

Perceived assignment and perceived efficacy: On direct questioning about pain control, responses were: CBD37 mg/mL—“No/No”; CBD17 mg/mL—“Yes/Yes”; placebo—“Yes” (one respondent; second did not answer).

When asked if they would use the oil they received again: CBD37 mg/mL—“Yes” from both respondents; CBD17 mg/mL—“Yes” (one respondent; second did not answer); placebo—“Yes” (one respondent; second did not answer).

These mixed responses underscore the importance of managing expectations and sensory matching the placebo to preserve blinding and reduce expectancy effects.

### 2.6. Safety and Tolerability

No serious adverse events occurred. Mild events (e.g., drowsiness, dry mouth) were infrequent and self-limiting. No participants discontinued treatment due to adverse effects in this pilot cohort.

## 3. Discussion

Given the very small sample (*n* = 8) and baseline imbalances, findings are descriptive and hypothesis-generating only. This pilot offers practice-oriented evidence that higher-concentration CBD (37 mg/mL) may yield clinically visible analgesia after simple tooth extraction, with pain trajectories mirroring standard non-opioid therapy (alternating ibuprofen/acetaminophen). By contrast, the lower concentration (17 mg/mL) was indistinguishable from placebo, suggesting a dose threshold for acute analgesia in this setting. Tolerability was favorable, supporting CBD’s potential as one component of multimodal, non-opioid dental pain management.

Two practical lessons emerged. First, although blinding was effective for CBD vs. placebo, participants could easily distinguish the TAU arm and note taste differences among oils. Future studies will incorporate a hemp-flavored, sensory-matched placebo to minimize expectancy bias and maintain blinding integrity. Second, EMA was feasible and informative; frequent in-the-moment ratings minimized recall bias and captured early pain dynamics.

Key limitations include the very small sample, open-label TAU arm, and absence of objective or biomarker endpoints. Adherence verification by bottle weight cannot confirm dosing schedule; future work will employ electronic monitoring or smart caps to strengthen adherence assessment. Taken together with preclinical data on CBD’s modulation of inflammatory signaling. Considering preclinical evidence that CBD modulates inflammatory signaling through CB_2_/TRP/5-HT_1_A/PPAR pathways, these pilot data justify a fully powered randomized trial incorporating (i) at least two CBD doses, (ii) a taste-matched placebo, (iii) EMA for high-resolution pain trajectories, and (iv) exploratory biomarker assays to clarify mechanisms. Safety conclusions have been tempered: tolerability appeared favorable within this small cohort, but larger samples are required to characterize risk and adverse event profiles. Our findings align with growing but heterogeneous evidence on cannabinoids for dental and orofacial pain. A Phase IIa randomized trial demonstrated that oral purified CBD reduced odontogenic pain in the emergency setting, with stronger effects at higher doses, supporting a dose–response relationship [17]. Conversely, a double-blind trial testing CBD-rich extract in endodontic patients reported no significant differences in postoperative pain or dental anxiety compared with placebo, highlighting variability across clinical contexts [19]. More consistent benefits has been observed in temporomandibular disorders, where topical and intraoral CBD significantly reduced pain intensity and muscle activity versus placebo in randomized trials [20]. Similarly, topical CBD accelerated healing and provided late-stage analgesia in patients with recurrent aphthous ulcers [21]. Together, these trials suggest that CBD may exert clinically meaningful analgesic effects for select orofacial pain states (e.g., acute odontogenic pain, some TMD-related pain), yet efficacy is not uniform across all dental indications. Heterogeneity in formulations, dosing regimens, and study designs likely contribute to mixed outcomes. Importantly, product quality and contamination risks remain a central threat to both internal validity and clinical translation, and dosing guidance is complicated by route-dependent bioavailability, food effects, and variable pharmacokinetics.

Lessons from these trials inform the design of future studies: Tolerability appeared favorable within this small cohort; however, larger and adequately powered studies are required to comprehensively characterize safety and risk profiles. Higher dosing strategies with pharmacokinetic verification, rigorous blinding with cannabis-flavor/taste-matched placebos, and high-frequency outcome sampling to capture early analgesic effects are critical for robust interpretation. In addition, mechanistic assays such as inflammatory biomarker profiling could clarify CBD’s analgesic pathways and help reconcile inconsistent findings. By integrating these methodological refinements, future work can better define the therapeutic role of CBD within multimodal, non-opioid approaches to dental pain management.

## 4. Materials and Methods

### 4.1. Study Design and Setting

This randomized clinical trial was conducted at the University of Tennessee Health Science Center (UTHSC) College of Dentistry’s Dunn/Delta Dental Clinic. Institutional Review Board approval was obtained (IRB# 20-07123-XP), and all participants provided written informed consent. The study adhered to the Declaration of Helsinki and Good Clinical Practice guidelines. This study was registered at ClinicalTrials.gov (Identifier: NCT04271917). The trial was sponsored by the University of Tennessee. The protocol specified a planned enrollment of 120 adults randomized equally to four arms (CBD17 mg/mL, CBD37 mg/mL, placebo, treatment-as-usual [TAU]). Due to the COVID-19 clinic closure, only a pilot cohort was accrued and is analyzed here.

### 4.2. Participants

**Figure 2:**
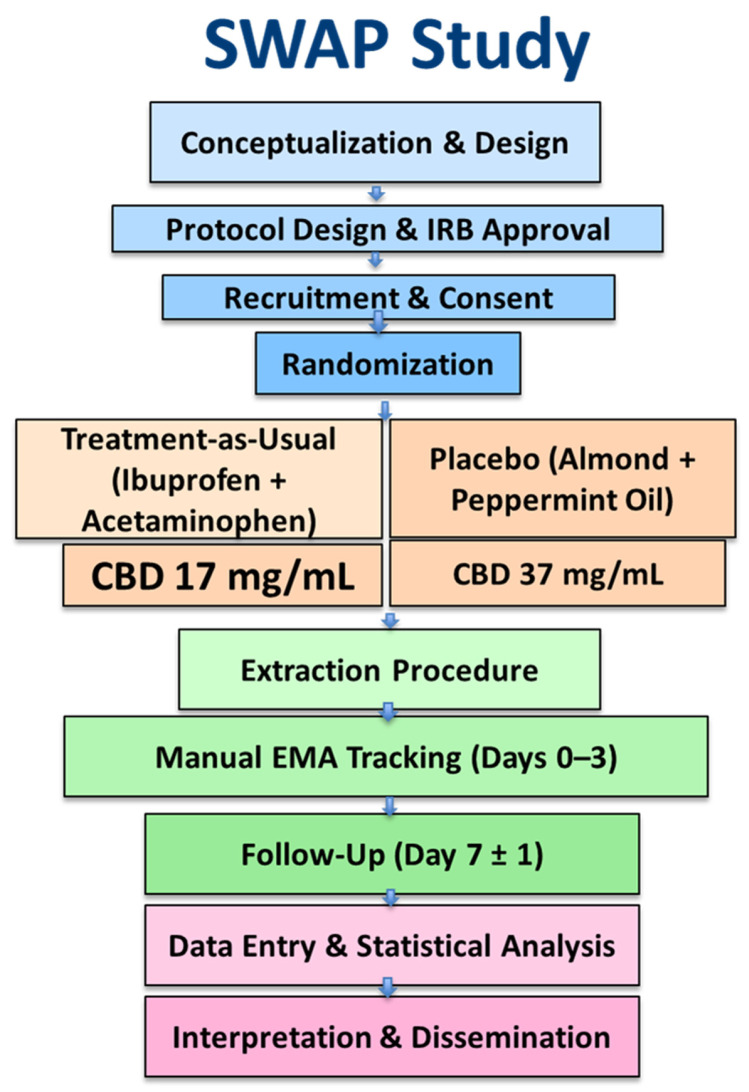
Study Design and Workflow of the SWAP Trial. Flowchart depicting the sequential phases of the Simple Tooth Extraction With Analgesic Phytocannabinoid (SWAP) study. Participants were randomized into four arms (treatment-as-usual, placebo, CBD 17 mg/mL, and CBD 37 mg/mL), completed 72-h post-extraction pain tracking via manual EMA logs, and returned for a 7-day follow-up to assess adherence and analgesic outcomes.

Eligible participants were ≥18 years, scheduled for simple (non-surgical) tooth extraction, and had no contraindications to CBD, almond oil, peppermint oil, hempseed oil, acetaminophen, or ibuprofen. Additional requirements included access to a cell phone and availability for a 7-day follow-up (Figure 2).

Exclusion criteria included pregnancy, uncontrolled systemic disease, known allergy to study medications/excipients, cannabis use within the prior 30 days, and concurrent participation in another interventional study.

Pilot enrollment: Prior to the pandemic-related shutdown, 11 patients were consented; 8 met eligibility and were randomized with equal allocation (*n* = 2 per arm: CBD 17 mg/mL, CBD 37 mg/mL, placebo, TAU).

### 4.3. Randomization and Blinding

Randomization used a computer-generated block design (blocks of 8 or 12). The randomization scheme was generated by the study statistician before recruitment began and made accessible to study staff via a shared Excel file. This file contained the allocation IDs as well as the arms, which were masked using a color code (e.g., green, red, yellow, or blue, where green = TAU, red = placebo, yellow = CBD17 mg/mL, and blue = CBD37 mg/mL). This design was double-blinded for three of the four arms. Because the TAU arm consisted of tablets rather than dropper vials, both the study staff and participants could tell if they were chosen for the TAU arm.

### 4.4. Interventions and Product Quality Control

CBD arms received a 7-day supply in dropper vials and were instructed to take 0.5 mL orally every 4–6 h as needed for pain. The placebo arm received almond/peppermint oil in identical vials, dosed identically. CBD oils were selected from a vendor providing third-party certificates of analysis (CBD concentration in mg/mL; screening for THC, microbials, terpenes, heavy metals, pesticides).

The TAU arm received blister packs of a combined ibuprofen (200 mg) and acetaminophen (325 mg) dose to be self-administered every 6–8 h.

All participants were instructed to reach out to the dental provider should breakthrough pain exceed an 8/10 on the pain rating scale.

### 4.5. Outcomes

The primary outcome was self-reported pain intensity during the first 72 h following tooth extraction. Pain was assessed using a 0–10 Numeric Rating Scale (NRS) administered via ecological momentary assessment (EMA) during waking hours (self-reported). Participants recorded each assessment on paper diaries provided at the time of extraction, which also included fields for adherence verification (oil weight or pill count) and concurrent pain ratings.

Secondary outcomes included:

- Worst post-operative pain intensity (NRS)
- Time to pain resolution (days from extraction to the first 24-h pain-free period)
- Treatment adherence (oil weight or pill count)
- Use of rescue analgesic medication (yes/no; number of doses)
- Adverse events (self-reported and investigator-verified)
- Qualitative feedback regarding taste, palatability, and perceived treatment assignment.

### 4.6. Ecological Momentary Assessment Procedures and Incentives

Paper diaries were provided to participants, with prompts scheduled at approximately 1.5-h intervals during waking periods for the first 3 days (maximum of 24 ratings per participant). Participants were instructed to complete as many diary entries as possible. Incentives included a gift card at baseline and follow-up for completing ≥75% of EMA entries (≥18 responses).

### 4.7. Follow-Up Visit

At day 7, participants returned for adherence verification (oil weight/pill count), completion of a feedback survey (satisfaction, perceived assignment), and adverse-event assessment.

### 4.8. Participant Payment

Participants received a $25 gift card to Kroger at the completion of the follow-up visit. Those who responded to 75% of the text messages received an additional $25 Kroger gift card.

### 4.9. Statistical Considerations

Given the pilot sample size (*n* = 8), inferential statistics were not emphasized; results are presented descriptively to inform design refinements and power calculations for the full trial.

## 5. Conclusions

This exploratory pilot study contributes preliminary feasibility evidence to the emerging yet mixed literature on cannabinoids for dental pain management. The findings indicate a potential dose-dependent analgesic effect favoring higher concentrations of CBD following tooth extraction, with favorable tolerability and the practical utility of real-time ecological momentary assessment (EMA) monitoring. However, given the small sample size and methodological limitations, the results remain inconclusive. Taken together with prior studies showing both positive and null outcomes across various dental and orofacial pain models, these findings highlight the need for larger, rigorously designed trials. Future research should incorporate optimized dosing strategies, taste-matched placebos, objective and clinician-rated outcomes, and biomarker profiling to better elucidate CBD’s role within multimodal, non-opioid approaches to acute dental pain.

## Figures and Tables

**Table 1:** Descriptive information.

		Medication Groups			
	Whole Sample	CBD 37 mg/mL	CBD 17 mg/mL	Placebo	Acetaminophen Ibuprofen
Race Caucasian	25%	0%	50%	0%	0%
Race African American	75%	100%	50%	100%	100%
Ethnicity Non-Latinx	100%				
Male	40%	100%	0%	0%	50%
Education Range	9th grade—graduate degree	12th grade—some college	12th grade—some college	12th grade—some college	9th grade—graduate degree
Average Pain Pre-Surgery	8.3	10	4	10	10

Descriptive characteristics of participants, including race, ethnicity, sex, education level, and average preoperative pain scores by treatment group. Notably, the CBD 17 mg/mL group reported lower average pre-surgical pain (4/10) compared with all other groups (10/10), a baseline imbalance acknowledged in Section 4.

**Table 2:** Response rate to ecological momentary assessment text prompts.

		Medication Groups			
	Whole Sample	CBD 37 mg/mL	CBD 17 mg/mL	Placebo	Acetaminophen Ibuprofen
Rate (number answered/24*100]	81.25%	79.17%	89.58%	66.67%	89.58%
Number answered average	19.5	19	21.5	16	21.5

Ecological Momentary Assessment (EMA) response rates over the first 72 h post-extraction. Participants received up to 24 text-based prompts. Three groups achieved ≥75% completion (≥18 responses), meeting the predefined feasibility threshold, while the placebo group averaged 66.7% (16 responses). No early discontinuations or protocol deviations occurred.

## Data Availability

The original contributions presented in this study are included in the article. Further inquiries can be directed to the corresponding authors.
